# A systematic review and network meta-analysis of cardiovascular safety of benzbromarone compared to febuxostat and allopurinol in patients with gout

**DOI:** 10.3389/fcvm.2025.1541307

**Published:** 2025-07-10

**Authors:** Ting-Syuan Lin, Yen-Liang Lin, Chiu-Hao Hsu, Song-Chou Hsieh, Wen-Yi Shau, Wei-Shiung Yang, Chi-Ling Chen

**Affiliations:** ^1^ Graduate Institute of Clinical Medicine, College of Medicine, National Taiwan University, Taipei, Taiwan; ^2^ Division of Allergy, Immunology and Rheumatology, Department of Internal Medicine, National Taiwan University Hospital Bei-Hu Branch, Taipei, Taiwan; ^3^ Division of Cardiology, Department of Internal Medicine, National Taiwan University Hospital Hsin-Chu Branch, Hsinchu, Taiwan; ^4^ Division of Neurosurgery, Department of Surgery, National Taiwan University Hospital, Taipei, Taiwan; ^5^ Division of Allergy, Immunology and Rheumatology, Department of Internal Medicine, National Taiwan University Hospital, Taipei, Taiwan; ^6^ Division of Endocrinology and Metabolism, Department of Internal Medicine, National Taiwan University Hospital, Taipei, Taiwan; ^7^ Department of Surgery, National Taiwan University Hospital, Taipei, Taiwan; ^8^ Graduate Institute of Epidemiology and Preventive Medicine, College of Public Health, National Taiwan University, Taipei, Taiwan

**Keywords:** febuxostat, allopurinol, benzbromarone, cardiovascular, gout

## Abstract

**Background:**

Gout is caused by hyperuricemia and is associated with cardiovascular diseases. Treatment for hyperuricemia primarily involves urate-lowering medications. Some trials showed higher cardiovascular mortality rates with febuxostat compared to allopurinol in gout patients. However, data on the cardiovascular safety of benzbromarone compared to allopurinol is limited, and there is no data comparing benzbromarone to febuxostat. This study aims to assess the cardiovascular safety of benzbromarone, febuxostat, and allopurinol in gout patients.

**Methods:**

A comprehensive search was conducted across PubMed and EMBASE from their inception to August 2024. Inclusion criteria were randomized controlled trials (RCTs) and cohort studies including adult patients with the diagnosis of gout, with urate-lowering medications. The outcome was the incidence of major adverse cardiovascular events. This systematic review and network meta-analysis were recorded in INPLASY with the ID INPLASY202460049.

**Results:**

A total of 176 studies were identified through the database search. There were 119 articles identified in EMBASE and 57 articles identified in PubMed. Following screening and review, 17 qualified studies (5 RCTs) were included in the network meta-analysis. The relative cardiovascular event risk (risk ratio, RR) for benzbromarone compared to febuxostat is 0.82 (95% CI 0.61–1.09), and for benzbromarone compared to allopurinol, the RR is 0.87 (95% CI 0.75–1.01). The RR for febuxostat compared to allopurinol is 1.08 (95% CI 0.97–1.20).

**Conclusion:**

Our network meta-analysis suggests a subtle trend indicating a lower risk of cardiovascular events for benzbromarone compared to both febuxostat and allopurinol in gout patients, although not statistically significant.

**Systematic Review Registration:**

https://inplasy.com/inplasy-2024-6-0049/, identifier INPLASY202460049.

## 1 Introduction

Gout is a metabolic disorder with hyperuricemia and urate crystal deposition followed by chronic inflammation and recurrent flares of acute arthritis. It is associated with increased risks of developing cardiovascular disease. Patients with gout have greater risks of mortality and many comorbidities, including chronic kidney disease, cardiovascular diseases, hypertension, and type 2 diabetes mellitus in comparison to those without gout (1–3). Urate-lowering medications play the pivotal role in the management of gout. Among the medications, xanthine oxidase inhibitors, febuxostat and allopurinol, are prescribed worldwide, whereas uricosuric agent, benzbromarone, is more often administered in some East Asia countries (4).

A number of studies have focused on the cardiovascular effects of xanthine oxidase inhibitors such as allopurinol and febuxostat due to their capability to suppress xanthine oxidase activity and reduce oxidative stress (5–7). Allopurinol had shown effects of scavenging oxygen radicals, improving insulin sensitivity and anti-inflammatory actions (6, 7). Xanthine oxidase inhibitors have long been the pharmacologic candidate to modulate cardiovascular risk, based on their theoretically blockade of oxidative stress.

Benzbromarone is an uricosuric drug that enhances urate excretion by inhibiting urate transporter 1 (URAT1), the primary apical urate transporter in the kidney's proximal tubule. This inhibition decreases urate reabsorption and increases its excretion in the urine. Benzbromarone was never approved in the United States, and it was withdrawn from the European market in 2003 due to hepatotoxicity concerns. However, the drug remains widely used in the Asia-Pacific region (8).

The Cardiovascular Safety of Febuxostat and Allopurinol in Patients with Gout and Cardiovascular Morbidities (CARES) trial was a multicenter, double-blind, noninferiority trial involving patients with gout and cardiovascular disease. The result revealed noninferiority of developing adverse cardiovascular events between the febuxostat and the allopurinol group. But it demonstrated higher rates of all-cause and cardiovascular death in the febuxostat group (9). Thereafter, the U.S. Food and Drug Administration (FDA) issued warnings, indicating that febuxostat might increase cardiovascular mortality and all-cause mortality compared with allopurinol. The issues of cardiovascular safety about using urate-lowering medications have drawn great attention, and several studies have been conducted to assess this issue. The Febuxostat vs. Allopurinol Streamlined Trial (FAST) was another multicenter, prospective, open-label, non-inferiority trial to evaluate the safety issues of treatment among patients with gout. The result, on the contrary, showed that the febuxostat group was noninferior to the allopurinol group with respect to both the rate of composite adverse cardiovascular events and all-cause mortality (10). The conclusion that usage of febuxostat would increase the risk of cardiovascular death remains in doubt.

On the other hand, a potentially deadly side effect of using allopurinol has been well established in the past decades. This is a severe cutaneous adverse reaction induced by allopurinol, more frequently seen in patients with variant of HLA-B*58:01 allele. Unfortunately, the prevalence of HLA-B*58:01 is around 12%–20% in some Asian populations (11, 12). The risk of developing this serious adverse event seems high in these populations, making allopurinol undesirable as the first-line treatment for urate-lowering therapy and gout prevention. Comparing to allopurinol, febuxostat and benzbromarone are commonly prescribed in Taiwan (13). With regards to the cardiovascular safety of benzbromarone, there have been only two studies published. One study found benzbromarone was associated with a decreased cardiovascular risk compared to allopurinol; however, another study showed no significant difference in cardiovascular risk between allopurinol and benzbromarone (14, 15). There was no clinical trials directly comparing the cardiovascular safety outcomes between febuxostat and benzbromarone. Therefore, we conducted this network meta-analysis to evaluate the cardiovascular safety of benzbromarone, febuxostat, and allopurinol in gout patients.

## 2 Methods

The manuscript was prepared according to the Preferred Reporting Items for Systematic Reviews and Meta-Analyses (PRISMA) 2020 statement. The protocol of this study was registered and approved by INPLASY under the registration number of INPLASY202460049.

### 2.1 Eligibility criteria

This meta-analysis was based on the following PICO (population, intervention, comparison, outcome) to frame our study. The population included adult patients (≥18 years) with the diagnosis of gout. The intervention group was treated with benzbromarone, while the comparison group was treated with febuxostat or allopurinol. The outcome was the incidence of major adverse cardiovascular events, defined as a composite of cardiovascular death, non-fatal myocardial infarction, non-fatal stroke, and unstable angina with urgent coronary revascularization.

### 2.2 Information source and search strategy

PubMed and EMBASE were the main sources of electronic bibliographic database for literature search and were investigated from inception to August 2024. We used a combination of various terms for searching, including benzbromarone, febuxostat, allopurinol, cardiovascular, and gout as keywords. We only adopted articles written in English with full text available. Randomized clinical trials (RCTs) and comparative observational studies were both included. The types of studies such as animal studies, case series, case reports, reviews, abstracts, editorials, comments, and letters to the editor were not included in our meta-analysis.

### 2.3 Data collection

The data were extracted by an investigator according to the pre-defined PICO. The accuracy of extracted data was confirmed by another investigator. If the article did not provide adequate statistical parameters for analysis, such as hazard ratio or odds ratio, the article would not be included for network meta-analysis. Any discrepancies in the extracted data were identified and discussed in order to reach a consensus. The effect measures of interest included cardiovascular death, non-fatal myocardial infarction, non-fatal stroke, and unstable angina with urgent coronary revascularization. The data collected included the number of participants, country, and follow-up periods.

### 2.4 Risk of bias assessment

We applied Cochrane risk of bias tool to appraise the methodological quality. The domains included sequence generation, allocation sequence concealment, blinding of participants and personnel, blinding of outcome assessment, incomplete outcome data, selective reporting, and other biases. Assessment discrepancies were resolved by discussion until reaching consensus.

### 2.5 Statistical analysis

The network meta-analysis involved direct comparisons conducted using Review Manager (RevMan) Version 5.4 software (The Cochrane Collaboration, 2020) with a random-effects model, while indirect comparisons were performed using RStudio 2024.04.02 Build 764 and R 4.4.1 (2024-06-14 ucrt). The forest plots were generated for assessing the effects of individual study and pooled results. A funnel plot was created using Review Manager to illustrate the distribution of individual study effect and to evaluate the possibility of publication bias. A subgroup analysis of RCTs vs. observational trials was performed to evaluate the potential heterogeneity. A sensitivity analysis was performed to address the potential issue of overlapping populations. The hazard ratio with 95% confidence interval was used for the outcome of time-to-event data, and the odds ratio was used as a substitute if the hazard ratio was unavailable. All effect measures were converted to risk ratio for pooling results. *P* < 0.05 was considered as statistical significance. Cochran's Q test, with significance at *P* < 0.1, and *I*^2^ statistic, with 50% as substantial heterogeneity, were used to test the heterogeneity among the included studies.

## 3 Results

**Figure 1:**
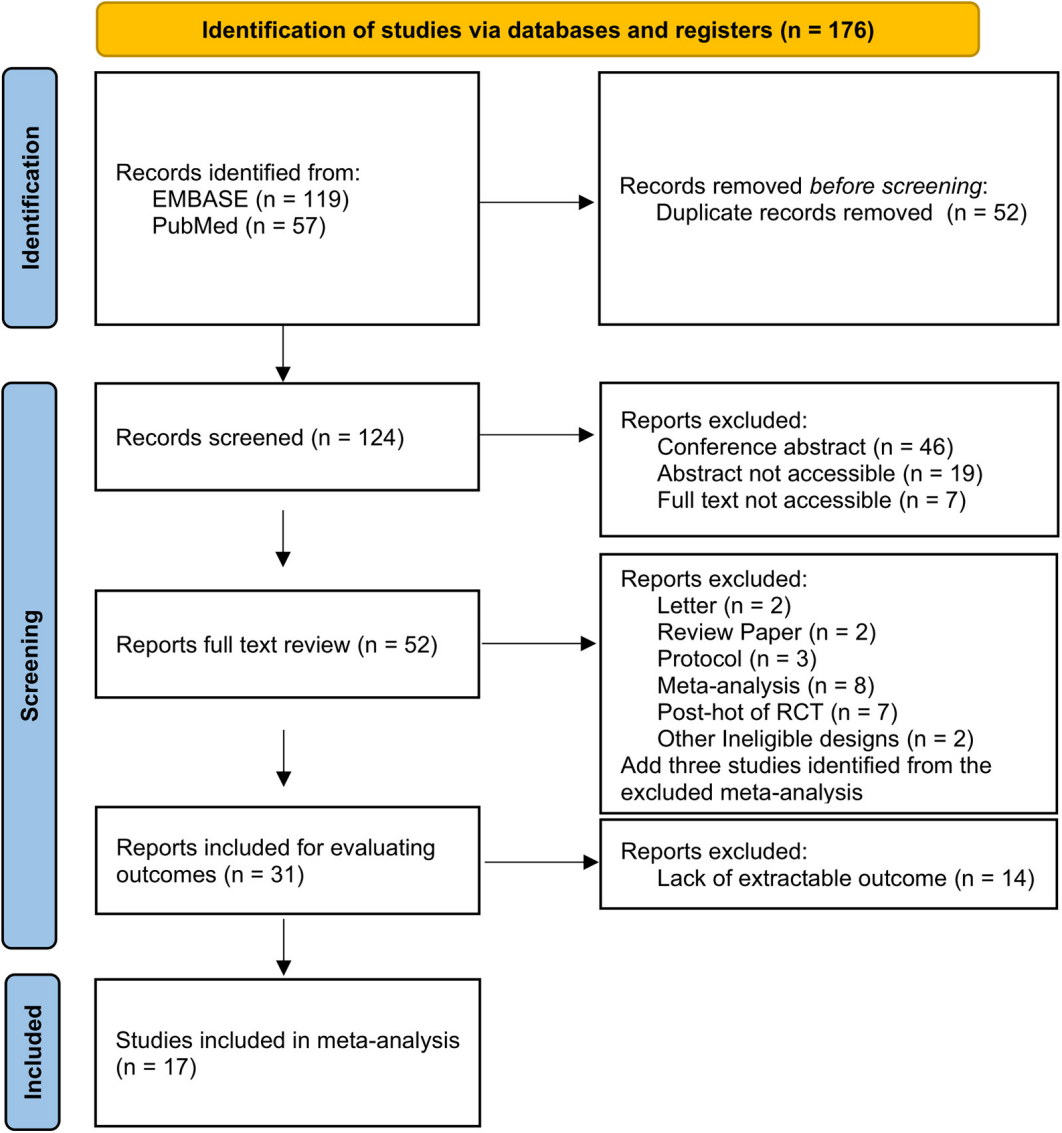
Flow diagram of the evidence search and selection.

**Figure 2:**
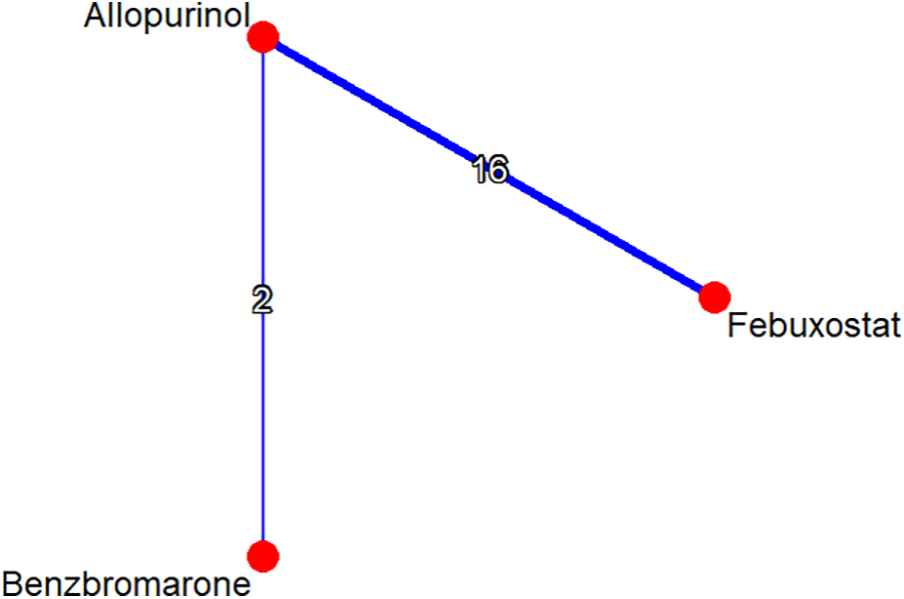
Network graph of studies according to febuxostat, benzbromarone, and allopurinol. The graph shows lines between treatments that have direct comparisons; thicker lines indicate that more comparison studies have been conducted.

A total of 176 studies were identified through the database search. There were 119 articles identified in EMBASE and 57 articles identified in PubMed. Following screening and review, 17 qualified studies were included in the network meta-analysis. The excluded studies were primarily conference abstracts, letters, review papers, protocols, meta-analyses, and *post-hoc* analyses of randomized controlled trials. Among the studies included, 12 cohort studies and 5 RCTs were adopted for further analysis. A flow diagram depicting the process of evidence search and selection is shown in Figure 1. Among one of the 12 cohort studies, the high cardiovascular risk population and low cardiovascular risk population were analyzed separately. The network graph of the studies according to febuxostat, benzbromarone, and allopurinol is shown in Figure 2.

The sample size of the studies ranged from 535 to 321,860 patients. The studies were conducted across different continents, including North America, Europe, and Asia. In total, there were 977,420 patients included in this analysis. Among them, there were 363,955 patients in the febuxostat group, 28,607 patients in the benzbromarone group, and 584,858 patients in the allopurinol group. All the five RCTs were febuxostat against allopurinol. Two observational studies were benzbromarone vs. allopurinol, whereas the rest ten were febuxostat vs. allopurinol. The characteristics of the included RCT studies are summarized in Table 1, and the characteristics of the included observational studies are summarized in Table 2.

**Table 1:** Summary of baseline characteristics for the included RCTs.

Author country	Study design	Participants included	Intervention	Follow-up periods	Outcomes
O’Dell et al. (2022) USA	RCT	472 vs. 468	Febuxostat vs. allopurinol	72 weeks	OR (95% CI)
(21)					0.98 (0.37–2.68)
Mackenzie et al. (2020) Europe	RCT	3,063 vs. 3,065	Febuxostat vs. allopurinol	A median of 6 years	HR (95% CI)
(10)					0.85 (0.70–1.03)
White et al. (2018) USA	RCT	3,098 vs. 3,092	Febuxostat vs. allopurinol	A median of 32 months	HR (95% CI)
(9)					1.03 (0.87–1.23)
Becher et al. (2010) USA	RCT	1,513 vs. 756	Febuxostat vs. allopurinol	6 months	OR (95% CI)
(22)					0.50 (0.07–3.73)
Schumacher et al. (2008) USA	RCT	267 vs. 268	Febuxostat vs. allopurinol	28 weeks	OR (95% CI)
(23)					5.10 (0.56–241.85)

**Table 2:** Summary of baseline characteristics for the included observational studies.

Author country	Sample size & study design	Participants included	Intervention	Follow-up periods	Outcomes
Jeong et al. (2023) Korea	Retrospective cohort	27,761 vs. 27,761	Febuxostat vs. allopurinol	A median of 467 days	HR (95% CI)
(24)		(1:1 match)			1.17 (1.10–1.24)
Shin et al. (2022) Korea	Retrospective cohort	160,930 vs. 160,930	Febuxostat vs. allopurinol	A mean of 250 days	HR (95% CI)
(25)		(1:1 match)			1.03 (0.95–1.12)
Weisshaar et al. (2022) Austria	Retrospective cohort	7,767 vs. 20,301	Febuxostat vs. allopurinol	A median of 640 days	HR (95% CI)
(26)					1.72 (1.59–1.89)
Cheng et al. (2022) Taiwan	Retrospective cohort	Low risk	Febuxostat vs. allopurinol	A median of 640 days vs. 907 days	HR (95% CI)
(27)		61,424 vs. 61,424			1.15 (1.07–1.24); HR (95% CI)
		High risk			
		12,795 vs. 12,795			1.26 (1.15–1.37)
		(1:1 match)			
Eun et al. (2022) Korea	Retrospective cohort	7,868 vs. 23,603	Benzbromarone vs. allopurinol	14,862 PYs vs. 6,705 PYs	HR (95% CI)
(14)		(1:3 match)			0.96 (0.77–1.20)
Kang et al. (2021) Korea	Retrospective cohort	20,739 vs. 103,695	Benzbromarone vs. allopurinol	A mean of 1.16 years	HR (95% CI)
(15)		(1:5 match)			0.82 (0.71–0.95)
Ju et al. (2020)	Retrospective cohort	276 vs. 828	Febuxostat vs. allopurinol	A median of 1.97 years	HR (95% CI)
Hong Kong		(1:3 match)			0.67 (0.42–1.09)
(28)					
Kang et al. (2019) Korea	Retrospective cohort	9,910 vs. 39,640	Febuxostat vs. allopurinol	A median of 32 months	HR (95% CI)
(29)		(1:4 match)			0.92 (0.76–1.11)
Chen et al. (2019) Taiwan	Retrospective cohort	5,262 vs. 5,257	Febuxostat vs. allopurinol	A mean of 2 years	HR (95% CI)
(30)		(1:1 match)			1.23 (0.98–1.56)
Su et al. (2019) Taiwan	Retrospective cohort	44,111 vs. 44,111	Febuxostat vs. allopurinol	A mean of 200 days vs. 160 days	HR (95% CI)
(31)		(1:1 match)			1.07 (0.97–1.18)
Zhang et al. (2018) USA	Retrospective cohort	24,936 vs. 74,808	Febuxostat vs. allopurinol	A mean of 1.1 years	HR (95% CI)
(32)		(1:3 match)			1.01 (0.94–1.08)
Foody et al. (2017) USA	Retrospective cohort	370 vs. 2,056	Febuxostat vs. allopurinol	A median of 9 months	HR (95% CI)
(33)					0.52 (0.30–0.91)

**Figure 3:**
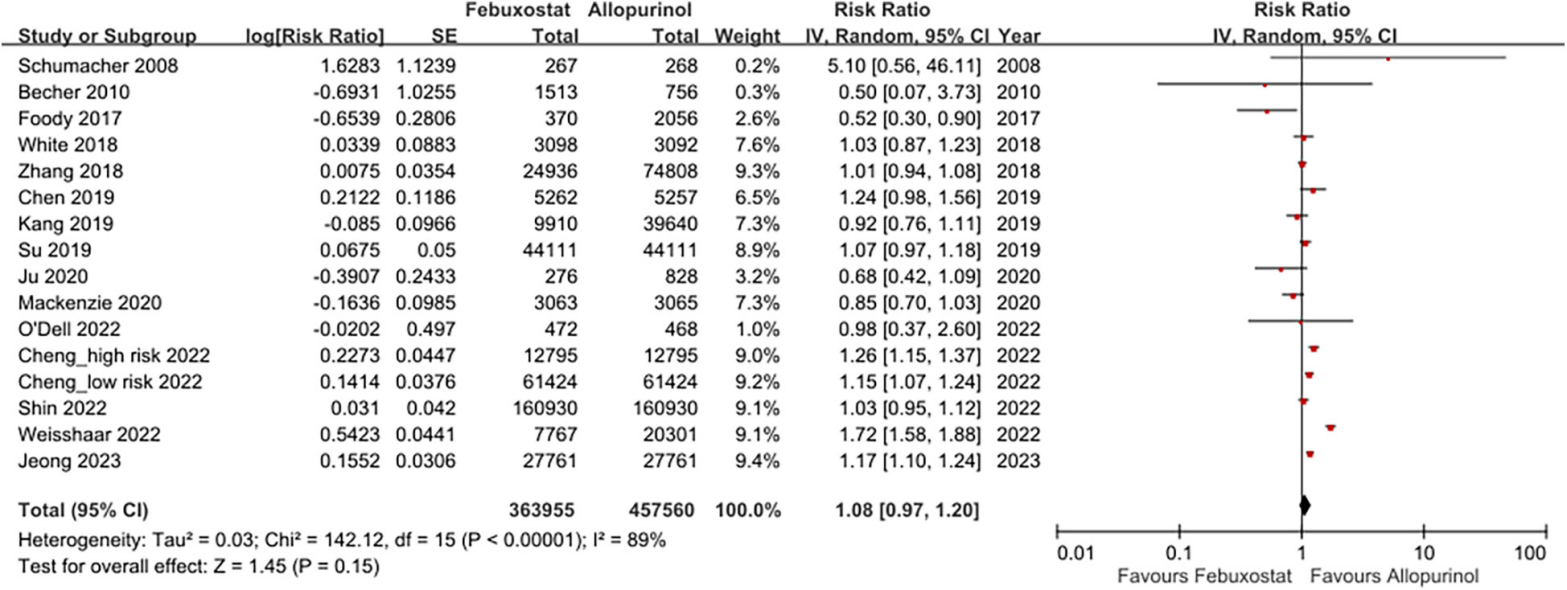
This forest plot provides a summary of the risk ratio for cardiovascular events of febuxostat and allopurinol across the included studies, highlighting both the individual and pooled effects.

**Figure 4:**
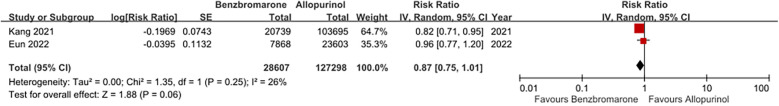
This forest plot provides a summary of the risk ratio for cardiovascular events of benzbromarone and allopurinol across the included studies, highlighting both the individual and pooled effects.

The forest plots illustrating cardiovascular safety of febuxostat, allopurinol, and benzbromarone is provided as Figures 3,4. The relative cardiovascular event risk (risk ratio, RR) for febuxostat compared to allopurinol is 1.08 (95% CI 0.97–1.20), and for benzbromarone compared to allopurinol the RR is 0.87 (95% CI 0.75–1.01). The league table with network meta-analysis estimates for cardiovascular outcomes is provided as Table 3. The analysis demonstrated that the relative cardiovascular event risk (RR) for benzbromarone compared to febuxostat is 0.82 (95% CI 0.61–1.09). The forest plot for cardiovascular safety of febuxostat, benzbromarone, and allopurinol using febuxostat as the reference is provided in Supplementary Figure S1.

**Table 3:** The league table compares relative cardiovascular event risk for each drug pair, including direct and indirect comparisons.

**Benzbromarone**		
0.88 (0.67; 1.15)	**Allopurinol**	
0.82 (0.61; 1.09)	0.93 (0.84; 1.03)	**Febuxostat**

Values are risk ratios (95% credible interval).

**Figure 5:**
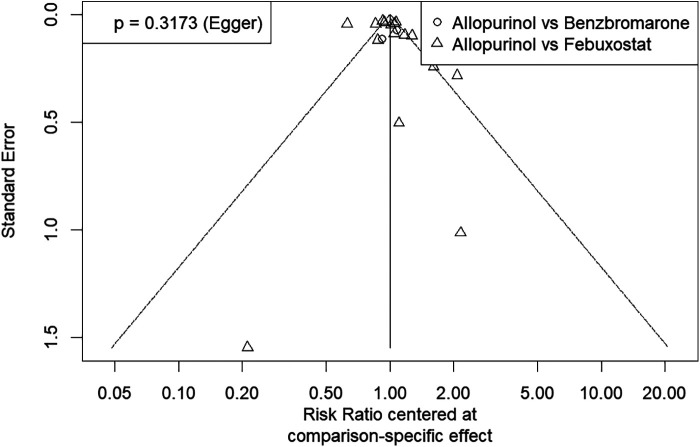
Funnel plot for assessing publication bias of studies included in this network meta-analysis. The symmetrical distribution of study effects depicted in this plot suggests a low likelihood of publication bias.

The funnel plot was used to assess publication bias in this network meta-analysis. The Egger test was performed, and the *P* values was 0.32. The plot shows that the effect estimates are distributed symmetrically, suggesting there is no strong evidence of publication bias. The funnel plot for assessing the studies included in this meta-analysis is provided in Figure 5.

In studies comparing the cardiovascular risks of febuxostat and allopurinol, there was high heterogeneity among these studies with *I*^2^ = 89%. We separated RCTs and observational studies for further subgroup analysis. In the RCTs, the RR of using febuxostat compared to allopurinol was 0.95 with 95% CI 0.80–1.12, and the heterogeneity of these RCTs was low as *I*^2^ = 18%. Among observational studies, the RR of using febuxostat compared to allopurinol was 1.11 (95% CI 0.99–1.24), and the heterogeneity among these observational studies with *I*^2^ = 92%. The forest plot illustrating this subgroup analysis is provided in Supplementary Figure S2.

Possible overlapping of populations in two cohort studies comparing benzbromarone and febuxostat was noted (14, 15). A sensitivity analysis was performed to address these concerns. After excluding one of the two studies, the analysis showed that excluding Eun et al. (2022) resulted in an RR of 0.76 (95% CI 0.52–1.11) for the difference in the risk of cardiovascular events between benzbromarone and febuxostat users, while excluding Kang et al. (2021) resulted in an RR of 0.89 (95% CI 0.59–1.35). The league table with network meta-analysis estimates for cardiovascular outcomes excluding one of the two studies is provided in Supplementary Table S1 and Table S2.

We evaluated the risk of bias in the studies using the Cochrane bias tool. Overall, we found a moderate to high risk of bias due to issues such as selection of participants and blinding of participants and outcome assessors. In order to minimize the impact of confounders, many observational studies used propensity score analysis. The bias risk assessments for each included study is summarized in Supplementary Figure S3. A graphical representation of each bias item presented as percentages is provided in Supplementary Figure S4.

## 4 Discussion

Treatment for hyperuricemia primarily involves low-purine diets and urate-lowering medications. The main types of urate-lowering medications are xanthine oxidase inhibitors (e.g., allopurinol and febuxostat) and uricosurics (e.g., benzbromarone). Taiwan is one of the few countries marketing benzbromarone, a drug not commonly available in the United States or European Union. Therefore, publications about benzbromarone especially in comparison to febuxostat are limited. Since benzbromarone is not a xanthine oxidase inhibitor, including benzbromarone in the studies on cardiovascular risk of urate-lowering medications may further clarify the cardiovascular risks associated with these treatments.

Gout and hyperuricemia are both cardiovascular risk factors. Urate-lowering medications, theoretically, can reduce the cardiovascular risk by lowering uric acid level and reduce gout activity. Furthermore, recent studies suggested that circulating xanthine oxidoreductase, independent of its enzymatic product of uric acid, is a novel biomarker associated with the progression of cardiovascular diseases (16–19).

Both xanthine oxidase inhibitors and uricosurics are effective for reducing levels of uric acid (8, 20). Different urate-lowering medications, such as xanthine oxidase inhibitors and uricosurics, might impact cardiovascular outcomes differently due to their varying mechanisms of action. If the cardiovascular benefits are primarily linked to reduced serum uric acid levels, then both uricosurics and xanthine oxidase inhibitors should theoretically improve cardiovascular outcomes. Conversely, if the cardiovascular advantages are resulted from antioxidant effects through xanthine oxidase inhibition, then uricosurics might not be as protective. Our study showed a lower risk of cardiovascular events for benzbromarone compared to both febuxostat and allopurinol in gout patients, although not yet reaching statistical significance. There should be more studies to evaluate the hypothesis that the antioxidant effects provided by xanthine oxidase inhibition are central to cardiovascular benefits.

The major limitation of our network meta-analysis was high heterogeneity. A subgroup analysis of RCTs vs. observational trials was performed to evaluate the potential source of heterogeneity. Selection bias, performance bias, detection bias, and attrition bias may also exist in the included cohort studies. Many cohort studies used health insurance databases, which may partially explain the presence of these biases.

## 5 Conclusion

Our network meta-analysis suggests a subtle trend indicating a lower risk of cardiovascular events for benzbromarone compared to both febuxostat and allopurinol in gout patients. Further direct comparative trials between benzbromarone and febuxostat/allopurinol are necessary to confirm and validate these findings.

## Data Availability

The original contributions presented in the study are included in the article/Supplementary Material, further inquiries can be directed to the corresponding author.
