# Supplementary figures and images for: A systematic review and network meta-analysis of cardiovascular safety of benzbromarone compared to febuxostat and allopurinol in patients with gout
Source: Front Cardiovasc Med. 2025 Jul 10;12:1541307. doi: 10.3389/fcvm.2025.1541307 (PMC12286930; doi:10.3389/fcvm.2025.1541307)

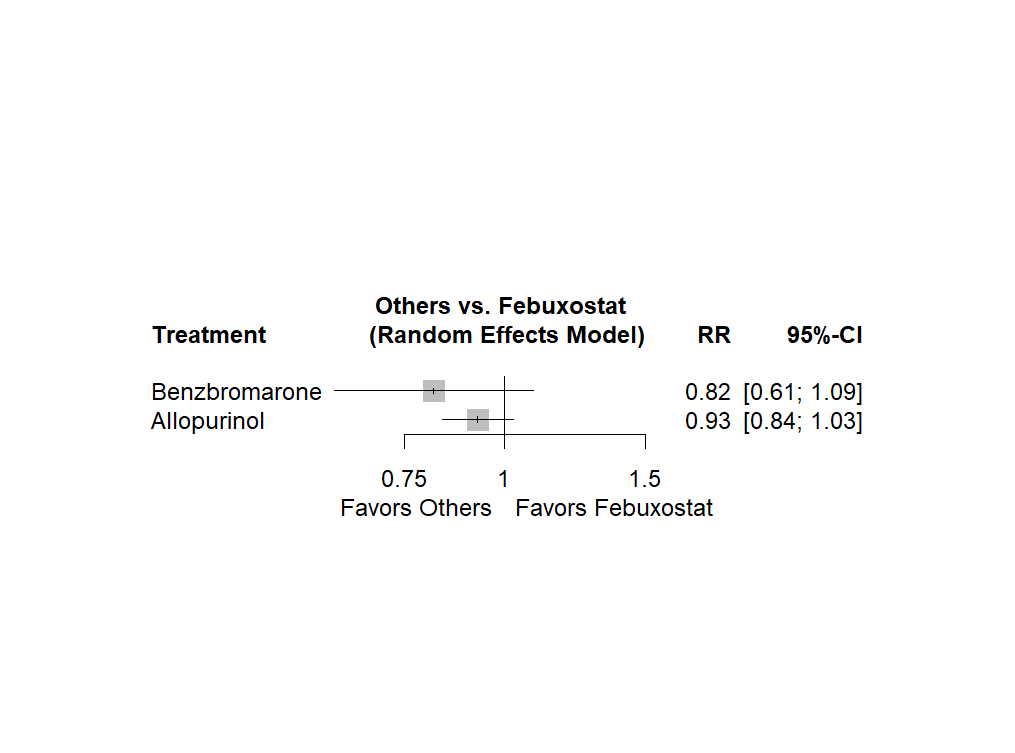

Supplement: Supplementary file 1 [file Image1.tiff]

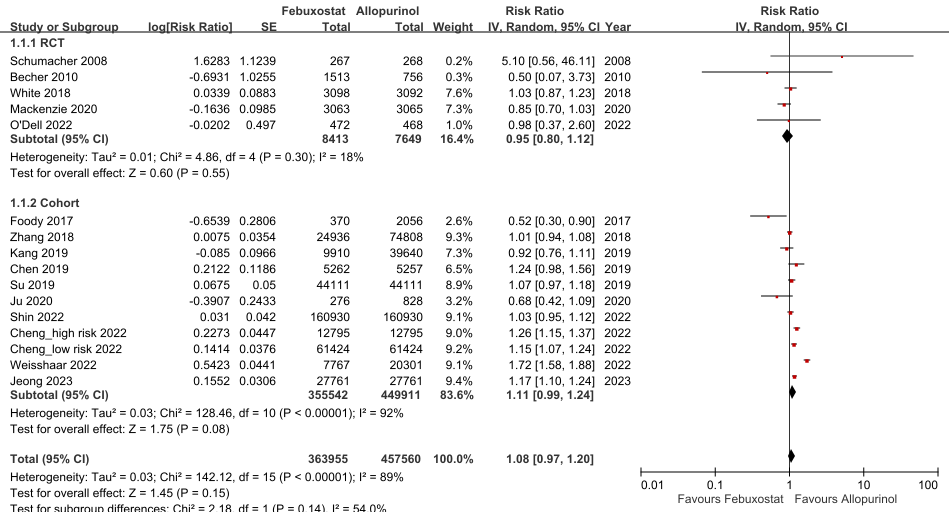

Supplement: Supplementary file 2 [file Image2.tif]

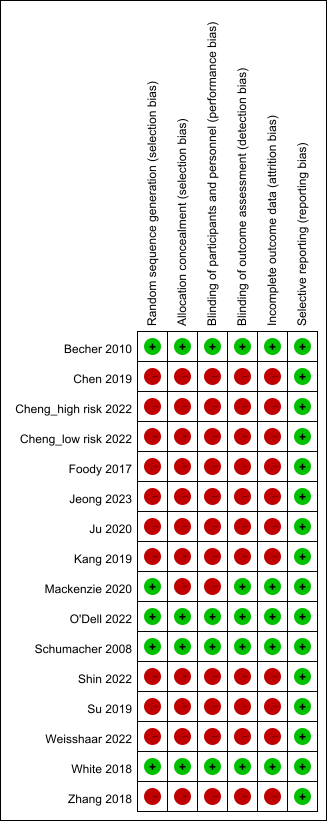

Supplement: Supplementary file 3 [file Image3.tif]

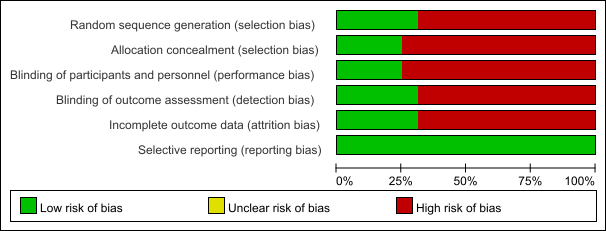

Supplement: Supplementary file 4 [file Image4.tif]
