# Supplementary material for: A systematic review and network meta-analysis of cardiovascular safety of benzbromarone compared to febuxostat and allopurinol in patients with gout
Source: Front Cardiovasc Med. 2025 Jul 10;12:1541307. doi: 10.3389/fcvm.2025.1541307 (PMC12286930; doi:10.3389/fcvm.2025.1541307)
Supplement: Supplementary file 5 [file Datasheet1.pdf]

Supplementary Table 1. The league table compares relative cardiovascular event risk for each drug pair, including direct and indirect comparisons (excluding Eun et al., 2022). Values are risk ratios (95% credible interval).

**Benzbromarone**		
**0.82 (0.57; 1.18)**	**Allopurinol**	
**0.76 (0.52; 1.11)**	**0.93 (0.84; 1.03)**	**Febuxostat**

Supplementary Table 2. The league table compares relative cardiovascular event risk for each drug pair, including direct and indirect comparisons (excluding Kang et al., 2021). Values are risk ratios (95% credible interval).

**Benzbromarone**		
**0.96 (0.64; 1.43)**	**Allopurinol**	
**0.89 (0.59; 1.35)**	**0.93 (0.84; 1.03)**	**Febuxostat**
